# Repetitive Passive Finger Movement Modulates Primary Somatosensory Cortex Excitability

**DOI:** 10.3389/fnhum.2018.00332

**Published:** 2018-08-20

**Authors:** Ryoki Sasaki, Shota Tsuiki, Shota Miyaguchi, Sho Kojima, Kei Saito, Yasuto Inukai, Naofumi Otsuru, Hideaki Onishi

**Affiliations:** Institute for Human Movement and Medical Sciences, Niigata University of Health and Welfare, Niigata, Japan

**Keywords:** passive movement, movement frequency, somatosensory-evoked potential, alpha oscillation, beta oscillation

## Abstract

Somatosensory inputs induced by repetitive passive movement (RPM) modulate primary motor cortex (M1) excitability; however, it is unclear whether RPM affects primary somatosensory cortex (S1) excitability. In this study, we investigated whether RPM affects somatosensory evoked potentials (SEPs) and resting state brain oscillation, including alpha and beta bands, depend on RPM frequency. Nineteen healthy subjects participated in this study, and SEPs elicited by peripheral nerve electrical stimulation were recorded from the C3’ area in order to assess S1 excitability (Exp. 1: *n* = 15). We focused on prominent SEP components such as N20, P25 and P45-reflecting S1 activities. In addition, resting electroencephalograms (EEGs) were recorded from C3’ area to assess the internal state of the brain network at rest (Exp. 2: *n* = 15). Passive abduction/adduction of the right index finger was applied for 10 min at frequencies of 0.5, 1.0, 3.0, and 5.0 Hz in Exp. 1, and 1.0, 3.0, and 5.0 Hz in Exp. 2. No changes in N20 or P25 components were observed following RPM. The 3.0 Hz-RPM decreased the P45 component for 20 min (*p* < 0.05), but otherwise did not affect the P45 component. There was no difference in the alpha and beta bands before and after any RPM; however, a negative correlation was observed between the rate of change of beta power and P45 component at 3.0 Hz-RPM. Our findings indicated that the P45 component changes depending on the RPM frequency, suggesting that somatosensory inputs induced by RPM influences S1 excitability. Additionally, beta power enhancement appears to contribute to the P45 component depression in 3.0 Hz-RPM.

## Introduction

Various types of repetitive somatosensory inputs are capable of evoking neuroplastic changes in the primary motor cortex (M1). Indeed, previous studies have reported changes in motor-evoked potentials (MEPs) elicited by transcranial magnetic stimulation (TMS) over the M1 after a prolonged period of peripheral nerve electrical stimulation (Ridding et al., 2000; Kaelin-Lang et al., 2002; Sasaki et al., 2017a). Similarly, muscle vibration or water flow stimulation has been shown to modulate MEPs (Steyvers et al., 2003; Sato et al., 2015). These MEP changes induced by repetitive somatosensory inputs are believed to occur at the level of the cortex, because neither H-reflex nor F-wave amplitude, which selectively reflects spinal motoneuron excitability, differs following peripheral nerve electrical stimulation (Ridding et al., 2000; Tinazzi et al., 2005; Golaszewski et al., 2012). In addition, cortical facilitatory and inhibitory circuits using a paired-pulse TMS paradigm are modulated by these stimulations (Mileva et al., 2009; Golaszewski et al., 2012; Sato et al., 2015).

Passive movement of the limbs can elicit somatosensory inputs in the same way that peripheral nerve electrical stimulation, muscle vibration and water flow stimulation do. Our previous studies showed that somatosensory inputs induced by repetitive passive movement (RPM) of the index finger for 10 min decreases M1 excitability (Miyaguchi et al., 2013; Otsuka et al., 2017; Sasaki et al., 2017b), depending on passive movement frequency (Sasaki et al., 2017b). In addition, we showed that MEPs decreased immediately after 0.5 Hz- and 1.0 Hz-RPM, while 5.0 Hz-RPM induced a decrease that lasted for 15 min. In contrast, 3.0 Hz-RPM had no effect on MEPs (Sasaki et al., 2017b). Thus, RPMs may contribute to a rehabilitation approach in order to induce neuroplastic change in M1; however, the M1 excitability depression mechanism induced by RPM remains unclear.

The primary somatosensory cortex (S1) is the main brain area that receives somatosensory input from various body parts (Kaas, 2004) and is closely related to M1 (Zarzecki et al., 1978; Keller et al., 1991). Accordingly, S1 excitability changes affect M1 excitability (Schabrun et al., 2012; Jacobs et al., 2014; Tsang et al., 2014). Therefore, we sought to use physiological data to determine as to whether RPMs affect S1 excitability. In addition, we further explored M1 excitability change mechanisms induced by RPM.

We used somatosensory-evoked potentials (SEPs) elicited by peripheral nerve electrical stimulation to assess S1 excitability. SEPs are comprised of plural components such as N20, P25 and P45. The N20 component of SEPs has been shown to elicit a response in area 3b of S1 following median nerve stimulation (Allison et al., 1991; Namiki et al., 1996). The current generation source for the P25 and P45 components is still debated; however, previous studies have reported P25 components in response to activation in area 1 and 2 of S1 and area 4 of M1 (Dinner et al., 1987), or area 1 of S1 (Allison et al., 1989a). In addition, the P45 component includes the activation in S1 (Allison et al., 1989b, 1992; Bufalari et al., 2007). We believed that it can non-invasively evaluate whether RPMs affect S1 excitability by analyzing these SEP components. Neuroimaging studies using magnetoencephalography have shown that the resting state brain network, including alpha (8–12 Hz) or beta (12–25 Hz) oscillations, relates to S1 and M1 activities (Ploner et al., 2006; Hall et al., 2011; Rossiter et al., 2014). These spontaneous neural activities temporally change with somatosensory stimulation (Gaetz and Cheyne, 2006; Houdayer et al., 2006; Müller-Putz et al., 2007; Enatsu et al., 2014). Thus, we considered that alpha and beta oscillations may be used to evaluate in detail the effect of somatosensory input induced by RPM on brain activity. In the present study, we investigated the effects of different RPM frequencies to test if somatosensory inputs induced by RPMs affect SEPs and spontaneous oscillations, including alpha and beta frequencies.

## Materials and Methods

### Subjects

Nineteen healthy subjects (13 males and 6 females; mean ± standard deviation, 22.4 ± 2.6 years; age range, 20–30 years) participated in this study. All subjects were right-handed with no history of neurological or psychiatric disorders. All subjects gave written informed consent to the experimental procedures. This study complied with the Helsinki declaration on human experimentation and was approved by the ethics committee of Niigata University of Health and Welfare.

### SEP Recordings Evoked by Peripheral Nerve Electrical Stimulation

Subjects sat in a comfortable reclining chair with a mounted headrest during experiments. This experiment was performed in a shielded room (Tokin Ltd, Sendai, Japan). Electroencephalogram (EEG) data were sampled at 10 kHz using an A/D converter (AIO AD16-16 (PCI)E, CONTEC, Osaka, Japan) and were amplified (BioTOP 6R12, NEC San-ei, Tokyo, Japan), band-pass filtered (0.5–3,000 Hz), and stored on a personal computer for later off-line analysis. EEG data were recorded from the C3’ area (2.0 cm posterior to C3 area) position of the international 10-20 system via Ag/AgCl electrodes (1.0 cm diameter). A reference electrode was placed at the Fz position, as this position can reduce mixing noise (Sonoo et al., 1996; Exp. 1). However, the frontal component mix for the active electrode used the Fz reference electrode (Desmedt and Cheron, 1981). Thus, we adopted the left earlobe (A1) and Fz reference electrodes, and the C3’ and Fz active electrodes were used to confirm that no SEPs changed with the activation of the frontal component in the reference electrode (Exp. 2). The earth electrode was placed on the Cz position in Exp. 1 and 2. Electrode skin impedance was always less than 10 kΩ. Electrical stimulation was applied to the right ulnar nerve at the wrist through a bar electrode, with the cathode positioned proximally delivering 0.2-ms square wave constant current pulses generated by a SEN-8203 stimulator (Nihon Kohden, Tokyo, Japan) to evoke SEPs. SEPs (360) were recorded from the active electrodes at an inter-stimulus interval of 1.5 s, with stimulus intensity set to 110% of the motor threshold at rest with the eyes opened. The motor threshold was determined as the minimum stimulus intensity that elicited M-waves to the right ulnar nerve of the wrist.

### EEG Recordings in the Resting Condition

Subjects sat in a comfortable reclining chair with a mounted headrest during experiments. This experiment was performed in a shielded room (Tokin Ltd, Sendai, Japan). EEG data were sampled at 2,000 Hz using an A/D converter (Power Lab 8/30, AD Instruments, Colorado Springs, CO, USA), amplified (BioTOP 6R12, NEC San-ei, Tokyo, Japan), low-pass filtered (70 Hz) and then stored on a personal computer for later off-line analysis. EEG data were recorded from the C3’ area (2.0 cm posterior to C3) and Fz positions of the international 10–20 system via Ag/AgCl electrodes (1.0 cm diameter). Two reference electrodes were placed on the A1 and Fz positions. The earth electrode was placed on the Cz position and the electro-oculogram data were recorded from two additional electrodes above and below the left eye (Vossen et al., 2015). Electrode skin impedance was always less than 10 kΩ. Subjects were instructed to maintain the rest position with their eyes closed for 4 min.

### Passive Movement Task

**Figure 1:**
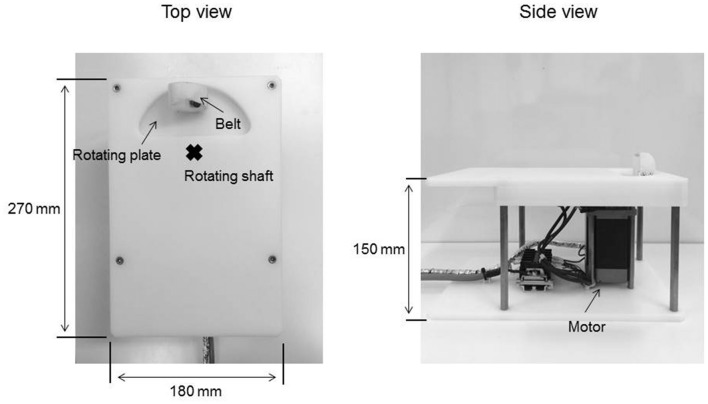
A custom-made device for passive movement.

The passive movement task was applied using a custom-made device consisting of a controller used in our previous study (Sasaki et al., 2017b) in order to set the movement velocity and range and a motor device to deliver the set passive movement sequence (Figure 1). The movement device consisted of a plastic plate, rotating plate and a motor. Subjects placed their right palm on the plastic plate, aligning the center of the metacarpophalangeal joint of the right index finger to the rotary shaft of the motor. The right index finger was fixed by a belt attached to the rotating plate and was moved passively in the abduction−adduction, axis from 0° to 20° abduction. The zero position was defined as the intermediate position of the metacarpophalangeal joint. In Exp. 1, RPM was performed for 10 min at 0.5 (20°/s), 1.0 (40°/s), 3.0 (120°/s) and 5.0 Hz (200°/s; 0.5, 1.0, 3.0 and 5.0 Hz-RPM). In Exp. 2, RPM was performed for 10 min at 1.0, 3.0, 5.0 Hz and control condition at rest (1.0, 3.0, 5.0 Hz-RPM and control). The explanation of the control condition is given below. We used a vibration dampener under the passive movement device to avoid vibratory stimulation.

### Kinematic Data

**Figure 2:**
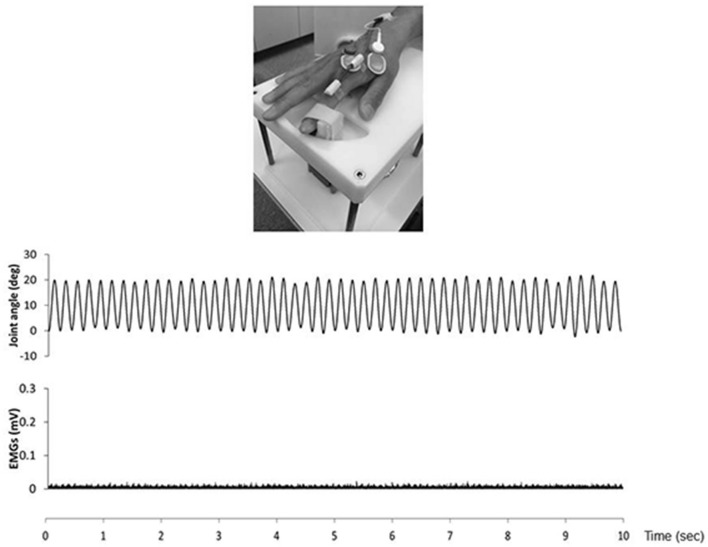
The top presence passive movement device. The middle presence joint angle during the 5.0 Hz-RPM. The under presence EMGs of the first dorsal interosseous (FDI) muscle during 5.0 Hz-RPM. Abbreviations: RPM, repetitive passive movement; EMG, electromyography.

We confirmed movement frequency and the joint angle during RPMs for subjects using an electrogoniometer (Single Axis Goniometer Type F 35, Biometrics Ltd, Newport, UK) attached to the metacarpophalangeal joint of right index finger (Figure 2). Surface electromyographic (EMG) activity was recorded from the right first dorsal interosseous (FDI) muscle via disposable Ag/AgCl electrodes (shape, oval; size, 44.3 mm × 22 mm; inter electrode distance, 10 mm) in a belly−tendon montage. EMG data were sampled at 4,000 Hz using an A/D converter (Power Lab 8/30, AD Instruments, Colorado Springs, CO, USA), amplified (100×; A-DL-720-140, 4 Assist, Tokyo, Japan), band-pass filtered (20–1,000 Hz), and then stored on a personal computer for later off-line analysis. Background EMG activity was monitored online from the right FDI muscle during RPM to confirm EMG activities, and subjects were instructed to relax if the root mean square background EMG activity exceeded 20 μV. However, background EMG and noise EMG activities were rarely observed (Figure 2).

### Exp. 1: SEP Recordings Before and After RPM

**Figure 3:**
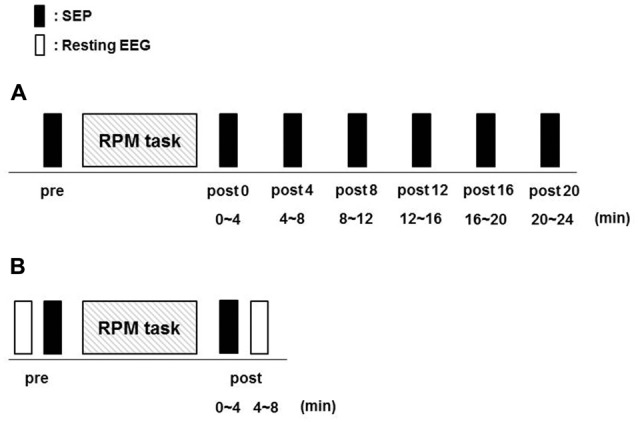
Experimental protocol. Fifteen subjects each participated in Exp. 1 and 2 to examine the effects of RPM conditions on SEPs and resting EEG, respectively. The minimum period between sessions for a single subject was 3 days. **(A)** In Exp. 1, SEPs were recorded 360 times at seven time-points (pre, post 0, post 4, post 8, post 12, post 16 and post 20) before and after RPM (0.5, 1.0, 3.0 and 5.0 Hz-RPM). **(B)** In Exp. 2, SEPs and resting EEG were recorded for 4 min at two time-points (pre and post) before and after Abbreviations: RPM (1.0, 3.0, 5.0 Hz-RPM and control). Abbreviations: RPM, repetitive passive movement; SEP, somatosensory-evoked potential; EEG, electroencephalogram.

Fifteen subjects (10 males and 5 females; mean ± standard deviation, 22.7 ± 2.8 years; range, 20–30 years) participated in Exp. 1. RPM was performed for 10 min at 0.5, 1.0, 3.0 and 5.0 Hz in random order on separate days, at least 3 days apart. SEPs (360) were recorded before (pre) RPM and then every 4 min for 24 min (post 0, post 4, post 8, post 12, post 16 and post 20) after RPM using the same electrical stimulation intensity (Figure 3A).

### Exp. 2: SEPs and Resting EEG Recordings Before and After RPM

Fifteen subjects (12 males and 3 females; mean ± standard deviation, 22.7 ± 2.5 years; range, 21–31 years) participated in Exp. 2. RPM was performed for 10 min at 1.0, 3.0, 5.0 Hz and control in random order on separate days, at least 3 days apart. SEPs and resting EEG data were recorded before (pre) RPM in order of resting EEG and SEPs, and then 4 min (post) after RPM in order of SEPs and resting EEG using the same electrical stimulation intensity (Figure 3B).

## Data Analysis

### Exp. 1

We used analysis software (EPLYZER II, KISSEI COMTEC, Nagano, Japan) for the analysis of SEPs. SEP recordings were acquired from 50 ms before to 200 ms after ulnar nerve electrical stimulation, and the average of 360 recordings was obtained during each time-point. Artifact detection was performed automatically with a threshold of 80 μV. The 20 ms period preceding stimulation was used as the baseline. The baseline-to-peak amplitudes (mean ± SE) of the three cortical components (N20, P25 and P45) from the C3’ area (Fz reference) were analyzed.

### Exp. 2

We performed SEP analysis in a similar method as Exp. 1, and the baseline-to-peak amplitudes (mean ± SE) of the three cortical components (N20, P25 and P45) from the C3’ area (Fz reference) were analyzed. The baseline-to-peak amplitudes (mean ± SE) of the two cortical components (P45 and frontal N30) were recorded from the C3’ area (A1 reference) and the Fz area (A1 reference) and were analyzed.

Resting EEG analyses were conducted using Matlab R2016b (Mathworks, Inc) and EEGLAB toolbox (Delorme and Makeig, 2004). The EEG data from the C3’ area (A1 reference), the C3’ area (Fz reference), and the Fz area (A1 reference) changed the sampling rate to 1,024 Hz from 2,000 Hz. Each of the EEG data for 4 min were segmented into 2 s-epochs (total of 120 epchs). Before analysis, artifact detection was performed automatically with a threshold of 80 μV, visually involving all EEG channels and electro-oculogram with the exclusion of all EEG segments that contained obvious or muscle artifacts. Additionally, fast Fourier transform for frequencies between 1 and 40 Hz was calculated for individual epoach of 2,048 samples (2 s) in 50% overlapping at 1,024 points using the Hanning window (time resolution 0.5 Hz). After fast Fourier transform, we analayzed the mean (mean ± SE) of the power spectral of the alpha (8–12 Hz) and beta (12–25 Hz) bands.

### Statistical Analysis

Statistical analysis was performed using PASW statistics software version 21 (SPSS; IBM, Armonk, NY, USA). In Exp. 1, two-way repeated-measures analysis of variance (RM-ANOVA) was applied to compare amplitudes and the latency of the three cortical components (N20, P25, and P45) from the C3’ area (Fz reference) with INTERVENTION (0.5, 1.0, 3.0 and 5.0 Hz-RPM) and TIME (pre, post 0, post 4, post 8, post 12, post 16 and post 20) as the main factors.

In Exp. 2, two-way RM-ANOVA with INTERVENTION (1.0, 3.0 and 5.0 Hz-RPM) and TIME (pre and post) was conducted on amplitudes and the latency of the three cortical components (N20, P25 and P45) from the C3’ area (Fz reference). In addition, two-way RM-ANOVA with INTERVENTION (1.0, 3.0 and 5.0 Hz-RPM) and TIME (pre and post) was conducted on amplitudes and the latency of the two cortical components (P45 and frontal N30) recorded from the C3’ area (A1 reference) and the Fz area (A1 reference). Two-way RM-ANOVA was conducted on the power spectral of the resting EEG, including the alpha and beta bands from the C3’ area (A1 reference and Fz reference) and the Fz area (A1 reference), and was compared with INTERVENTION (1.0, 3.0 and 5.0 Hz-RPM) and TIME (pre and post) as the main factors.

We calculated the effect size (partial *η^2^*) for all results of the two-way RM-ANOVA. The Mauchly’s test of sphericity was used to evaluate the sphericity assumption. If the sphericity assumption was violated, the Greenhouse–Geisser correction was conducted to adjust the *F*- and *p*-values. When a significant main effect or interaction was found, *post hoc* comparisons were conducted with Tukey’s test in Exp. 1 and paired *t*-tests in Exp. 2. In Exp. 2, Pearson’s correlation analysis was performed to determine whether RPM induced changes in P45 were associated with changes in the alpha and beta oscillations. Pearson’s correlation analysis was calculated using rate of change (P45 post/pre * 100) from the C3’ area (Fz reference) and (alpha or beta power post/pre * 100) from the C3’ area (A1 reference). Thus, we performed a control condition for RPM intervention to explore the correlation of the rate of change of P45 and resting EEG, including alpha and beta frequencies. A *p*-value< 0.05 was considered statistically significant for all the tests.

## Results

### Exp. 1: The Effects of RPM on SEPs From the C3’ Area (Fz Reference)

Table 1 presents the N20, P25 and P45 latency. Two-way RM-ANOVA for the latency of each component revealed no significant main effect of INTERVENTION or TIME and no interaction between INTERVENTION × TIME (Table 1).

**Table 1:** The results of latency for SEPs and two-way repeated-measures analysis of variance (RM-ANOVA) in Exp. 1.

Value of SEP latency		pre	post 0	post 4	post 8	post 12	post 16	post 20
N20 latency	0.5 Hz-RPM	18.5 ± 0.3	18.7 ± 0.3	18.8 ± 0.2	18.6 ± 0.3	18.8 ± 0.3	18.8 ± 0.3	18.8 ± 0.3
	1.0 Hz-RPM	19.0 ± 0.2	18.8 ± 0.2	18.7 ± 0.3	18.9 ± 0.2	18.9 ± 0.2	18.9 ± 0.3	19.0 ± 0.3
	3.0 Hz-RPM	18.8 ± 0.2	18.8 ± 0.2	18.9 ± 0.2	18.9 ± 0.2	18.9 ± 0.2	18.8 ± 0.2	18.9 ± 0.2
	5.0 Hz-RPM	18.7 ± 0.2	18.7 ± 0.2	18.8 ± 0.2	18.7 ± 0.4	18.7 ± 0.3	18.6 ± 0.3	18.7 ± 0.2
P25 latency	0.5 Hz-RPM	22.9 ± 0.5	23.3 ± 0.5	23.1 ± 0.4	23.2 ± 0.4	23.4 ± 0.5	23.3 ± 0.5	23.2 ± 0.5
	1.0 Hz-RPM	23.9 ± 0.5	23.9 ± 0.6	23.5 ± 0.5	23.1 ± 0.4	23.0 ± 0.5	23.4 ± 0.4	23.8 ± 0.6
	3.0 Hz-RPM	23.9 ± 0.7	23.3 ± 0.5	23.5 ± 0.5	23.7 ± 0.6	23.4 ± 0.5	23.1 ± 0.4	23.4 ± 0.5
	5.0 Hz-RPM	23.2 ± 0.5	23.8 ± 0.6	23.3 ± 0.6	23.3 ± 0.5	23.4 ± 0.5	23.4 ± 0.6	23.1 ± 0.5
P45 latency	0.5 Hz-RPM	52.4 ± 3.3	50.9 ± 3.1	52.6 ± 3.0	52.0 ± 2.6	52.2 ± 2.5	52.4 ± 2.7	51.4 ± 2.7
	1.0 Hz-RPM	51.1 ± 2 8	49.4 ± 2.9	50.8 ± 2.9	50.3 ± 3.1	50.4 ± 2.8	50.0 ± 2.8	50.9 ± 2.9
	3.0 Hz-RPM	48.0 ± 2.3	46.4 ± 2.3	47.9 ± 2.3	47.5 ± 2.7	47.0 ± 2.5	46.2 ± 2.3	47.7 ± 2.6
	5.0 Hz-RPM	49.7 ± 2.9	50.4 ± 2.9	50.8 ± 3.2	51.4 ± 2.9	52.0 ± 2.8	50.2 ± 2.7	51.4 ± 2.5
**Two-way RM-ANOVA**			***F*-value**		***p*-value**		**Effect size**	
N20 latency	CONDITION		0.861_(3,42)_		0.469		0.058	
	TIME		0.538_(6,84)_		0.778		0.037	
	CONDITION × TIME		1.060_(18,252)_		0.393		0.070	
P25 latency	CONDITION		0.556_(3,42)_		0.647		0.038	
	TIME		1.201_(6,84)_		0.314		0.079	
	CONDITION × TIME		1.585_(18,252)_		0.064		0.102	
P45 latency	CONDITION		1.513_(3,42)_		0.225		0.098	
	TIME		1.045_(2.538,35.525)_		0.376		0.098	
	CONDITION × TIME		0.665_(18,252)_		0.844		0.045	

**Figure 4:**
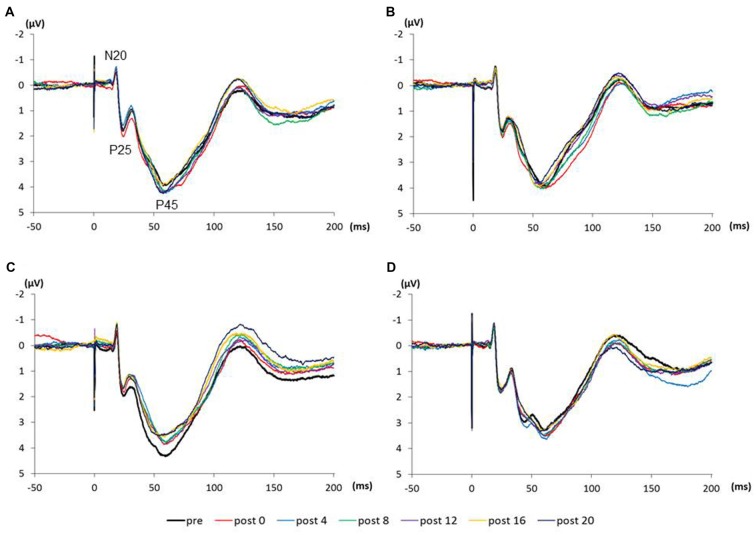
**(A)** 0.5 Hz-RPM, **(B)** 1.0 Hz-RPM, **(C)** 3.0 Hz-RPM, **(D)** 5.0 Hz-RPM. These SEP waveforms present the grand-averaged waveform (*n* = 15) from the C3’ area (Fz reference) before and after RPM. Abbreviations: RPM, repetitive passive movement.

**Figure 5:**
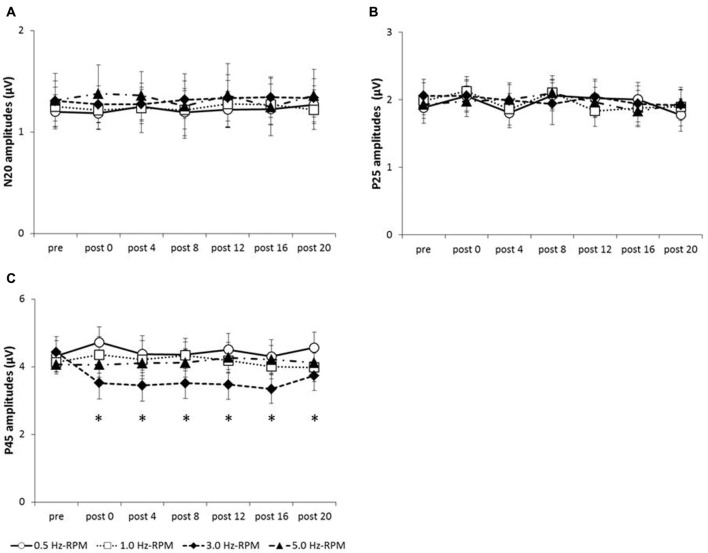
**(A)** N20 amplitudes, **(B)** P25 amplitudes, **(C)** P45 amplitudes. These graphs show the average of the N20, P25 and P45 amplitudes (mean ± SE) recorded at each time-point (pre, post 0, post 4, post 8, post 12, post 16 and post 20) before and after RPM (0.5, 1.0, 3.0 and 5.0 Hz-RPM). N20 and P25 amplitudes were not significant before and after RPM. P45 amplitudes decreased at post 0 to post 20 compared with the pre after the 3.0 Hz-RPM; however, other RPMs were not affected. *p < 0.05. Abbreviations: RPM, repetitive passive movement.

Figure 4 shows the SEP grand-averaged waveforms, whereas Figure 5 shows the SEP amplitudes for each component. Two-way RM-ANOVA for N20 amplitudes revealed no significant main effect of INTERVENTION (*F*_(1.492,20.890)_ = 0.168, *p* = 0.783, partial *η*^2^ = 0.012) or TIME (*F*_(6,84)_ = 0.240, *p* = 0.962, partial *η*^2^ = 0.017), and no interaction between INTERVENTION × TIME (*F*_(18,252)_ = 0.182, *p* = 1.000, partial *η*^2^ = 0.013). Similarly, no significant main effect of INTERVENTION (*F*_(3,42)_ = 0.037, *p* = 0.990, partial *η*^2^ = 0.098) or TIME (*F*_(3.356,46.986)_ = 0.494, *p* = 0.453, partial *η*^2^ = 0.061) and no interaction of INTERVENTION × TIME (*F*_(18,252)_ = 0.494, *p* = 0.960, partial *η*^2^ = 0.034) were observed at P25 amplitude. Additionally, no significant main effect of INTERVENTION (*F*_(3,42)_ = 1.119, *p* = 0.352, partial *η*^2^ = 0.074) or TIME (*F*_(2.624,36.740)_ = 0.709, *p* = 0.535, partial *η*^2^ = 0.048), but did reveal an interaction of INTERVENTION × TIME (*F*_(18,252)_ = 1.828, *p* = 0.023, partial *η*^2^ = 0.115) were observed at P45 amplitude. *Post hoc* analysis showed a significant decrease in P45 amplitudes from post 0-post 20 after the end of the 3.0 Hz-RPM compared with the pre (*p* < 0.05). No significant differences in P45 amplitudes between pre and post 0 to post 20 after 0.5, 1.0 and 5.0 Hz-RPM were observed.

### Exp. 2: The Effects of RPM on SEPs From the C3’ Area (Fz and A1 Reference) and Fz Area (A1 Reference)

Table 2 presents the N20, P25 and P45 latency from the C3’ area (Fz reference), the P45 latency from the C3’ area (A1 reference), and the frontal N30 latency from the Fz area (A1 reference). Two-way RM-ANOVA for the cortical components latency revealed no significant main effect of INTERVENTION or TIME, and no interaction between INTERVENTION × TIME.

**Table 2:** The results of latency for SEPs and two-way RM-ANOVA in Exp. 2.

Value of SEP latency			pre		post	
C3’	N20 latency	1.0 Hz-RPM	19.2 ± 0.23		19.1 ± 0.24
(Fz reference)		3.0 Hz-RPM	19.0 ± 0.26		19.2 ± 0.25
		5.0 Hz-RPM	18.9 ± 0.27		19.1 ± 0.24
	P25 latency	1.0 Hz-RPM	23.3 ± 0.36		23.1 ± 0.40
		3.0 Hz-RPM	23.1 ± 0.45		23.1 ± 0.46
		5.0 Hz-RPM	22.5 ± 0.31		22.9 ± 0.43
	P45 latency	1.0 Hz-RPM	44.1 ± 1.97		43.6 ± 1.94
		3.0 Hz-RPM	43.3 ± 1.90		42.7 ± 2.00
		5.0 Hz-RPM	42.7 ± 1.90		42.2 ± 2.30
C3’	P45 latency	1.0 Hz-RPM	43.8 ± 1.91		42.7 ± 1.81
(A1 reference)		3.0 Hz-RPM	41.4 ± 1.34		41.1 ± 1.42
		5.0 Hz-RPM	40.3 ± 1.76		40.8 ± 1.82
Fz	Frontal N30 latency	1.0 Hz-RPM	29.7 ± 0.93		29.6 ± 1.21
(A1 reference)		3.0 Hz-RPM	29.8 ± 1.02		28.9 ± 0.98
		5.0 Hz-RPM	29.5 ± 0.95		29.4 ± 1.19
**Two-way RM-ANOVA**			***F*-value**	***p*-value**	**Effect size**
C3′	N20 latency	CONDITION	1.474_(2,28)_	0.246	0.095
(Fz reference)		TIME	1.188_(1,14)_	0.294	0.078
		CONDITION × TIME	2.798_(1.436,20.104)_	0.098	0.167
	P25 latency	CONDITION	2.720_(2,28)_	0.083	0.163
		TIME	0.401_(1.14)_	0.537	0.028
		CONDITION × TIME	0.996_(2,28)_	0.382	0.066
	P45 latency	CONDITION	0.315_(2,28)_	0.732	0.022
		TIME	1.231_(1,14)_	0.286	0.081
		CONDITION × TIME	0.013_(2,28)_	0.987	0.001
C3′	P45 latency	CONDITION	1.151_(2,28)_	0.331	0.076
(A1 reference)		TIME	0.926_(1,14)_	0.352	0.062
		CONDITION × TIME	1.786_(2,28)_	0.186	0.113
Fz	Frontal N30 latency	CONDITION	0.116_(1.334,18.678)_	0.808	0.008
(A1 reference)		TIME	1.106_(1,14)_	0.311	0.073
		CONDITION × TIME	0.482_(2,28)_	0.622	0.033

Table 3 presents the SEP amplitudes for each component. N20, P25, and frontal N30 amplitudes revealed no significant main effect of INTERVENTION or TIME, and no interaction between INTERVENTION × TIME. Two-way RM-ANOVA for the P45 amplitude from the C3’ area (Fz reference) revealed no significant main effect of INTERVENTION, but did reveal an interaction with TIME and an interaction between INTERVENTION × TIME (*p* < 0.05). Additionally, Two-way RM-ANOVA for the P45 amplitude from the C3’ area (A1 reference) revealed no significant main effect of INTERVENTION or TIME, but did reveal an interaction between INTERVENTION × TIME (*p* < 0.05). *Post hoc* analysis showed a significant decrease in P45 amplitudes from the C3’ area (Fz reference and A1 reference) at post after the end of the 3.0 Hz-RPM compared with the pre (*p* < 0.05), while there were no significant differences in P45 amplitudes between pre and post after 1.0 and 5.0 Hz-RPM.

**Table 3:** The results of amplitudes for SEPs and two-way RM-ANOVA in Exp. 2.

Value of SEP amplitude			pre		post
C3’	N20 amplitude	1.0 Hz-RPM	1.58 ± 0.29		1.55 ± 0.26
(Fz reference)		3.0 Hz-RPM	1.56 ± 0.25		1.68 ± 0.28
		5.0 Hz-RPM	1.62 ± 0.14		1.51 ± 0.21
	P25 amplitude	1.0 Hz-RPM	2.55 ± 0.30		2.56 ± 0.37
		3.0 Hz-RPM	2.64 ± 0.33		2.48 ± 0.35
		5.0 Hz-RPM	2.74 ± 0.42		2.80 ± 0.49
	P45 amplitude	1.0 Hz-RPM	3.16 ± 0.35		3.51 ± 0.44
		3.0 Hz-RPM	3.48 ± 0.31		2.59 ± 0.25^*^
		5.0 Hz-RPM	3.23 ± 0.43		3.14 ± 0.33
C3’	P45 amplitude	1.0 Hz-RPM	2.68 ± 0.33		2.51 ± 0.31
(A1 reference)		3.0 Hz-RPM	2.80 ± 0.34		1.89 ± 0.24^*^
		5.0 Hz-RPM	2.48 ± 0.25		2.62 ± 0.42
Fz	Frontal N30 amplitude	1.0 Hz-RPM	1.84 ± 0.20		2.04 ± 0.25
(A1 reference)		3.0 Hz-RPM	2.03 ± 0.27		2.05 ± 0.30
		5.0 Hz-RPM	2.17 ± 0.29		1.97 ± 0.19
**Two-way RM-ANOVA**			***F*-value**	***p*-value**	**Effect size**
C3’	N20 amplitude	CONDITION	0.043_(2,28)_	0.958	0.003
(Fz reference)		TIME	0.035_(1,14)_	0.853	0.003
		CONDITION × TIME	0.728_(2,28)_	0.492	0.049
	P25 amplitude	CONDITION	0.601_(2,28)_	0.555	0.041
		TIME	0.031_(1,41)_	0.863	0.002
		CONDITION × TIME	0.370_(2,28)_	0.694	0.026
	P45 amplitude	CONDITION	0.254_(2,28)_	0.777	0.018
		TIME	5.977_(1,14)_	0.028	0.299
		CONDITION × TIME	3.418_(2,28)_	0.047	0.196
C3’	P45 amplitude	CONDITION	0.442_(2,28)_	0.647	0.031
(A1 reference)		TIME	4.111_(1,14)_	0.062	0.227
		CONDITION × TIME	3.890_(2,28)_	0.032	0.217
Fz	Frontal N30 amplitude	CONDITION	0.402_(1.306,18.288)_	0.589	0.028
(A1 reference)		TIME	0.001_(1,14)_	0.972	<0.001
		CONDITION × TIME	0.784_(1.427,19.983)_	0.430	0.053

^*^p < 0.05.

### Exp. 2: The Effects of RPM on Resting EEG From the C3’ Area (Fz and A1 Reference) and Fz Area (A1 Reference)

**Figure 6:**
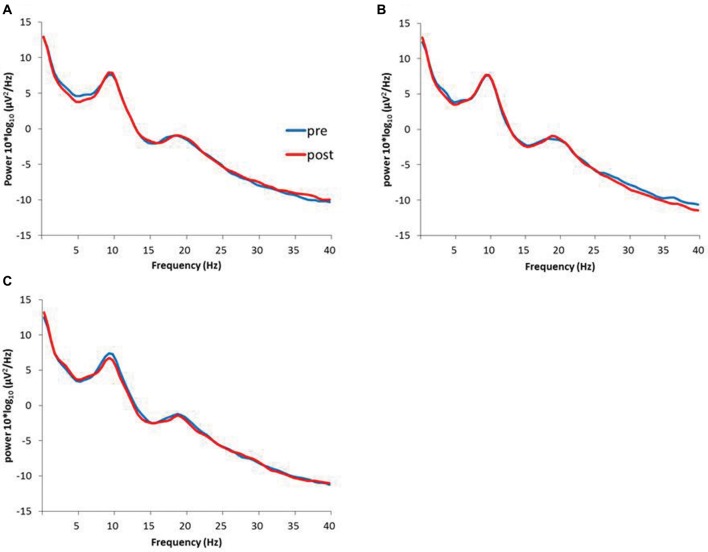
**(A)** 1.0 Hz-RPM, **(B)** 3.0 Hz-RPM, **(C)** 5.0 Hz-RPM. These waveforms present the grand average of the resting EEG (1–40 Hz; *n* = 15) from the C3’ area (A1 reference) before and after RPM. Abbreviations: RPM, repetitive passive movement; EEG, electroencephalogram.

**Figure 7:**
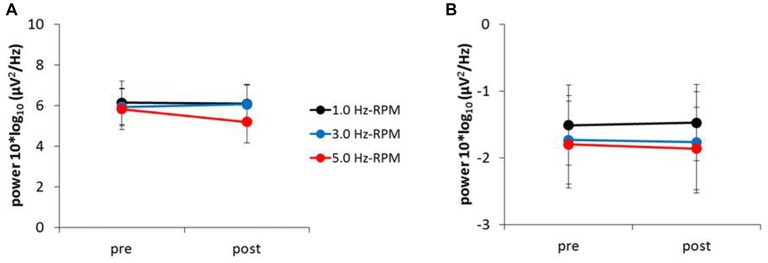
**(A)** Alpha power, **(B)** beta power. These graphs show the average of the alpha and beta powers (mean ± SE) recorded from the C3’ area (A1 reference) for each time-point (pre and post) before and after RPM (1.0, 3.0 and 5.0 Hz-RPM). Alpha and beta powers were not significant before and after RPM. Abbreviations: RPM, repetitive passive movement.

Figure 6 presents the resting EEG grand-averaged power spectrum, whereas Figure 7 presents the power spectrum of alpha and beta band frequencies at pre and post. Two-way RM-ANOVA for these oscillations revealed no significant main effect of INTERVENTION or TIME, and no interaction between INTERVENTION × TIME (Table 4).

**Table 4:** The results of two-way RM-ANOVA for alpha and beta power in Exp. 2.

			*F*-value	*p*-value	Effect size
C3′	Alpha power	CONDITION	0.781_(2,28)_	0.468	0.053
(A1 reference)		TIME	0.569_(1,14)_	0.463	0.039
		CONDITION × TIME	1.006_(2,28)_	0.378	0.067
	Beta power	CONDITION	0.915_(2,28)_	0.412	0.061
		TIME	0.044_(1,14)_	0.837	0.003
		CONDITION × TIME	0.057_(2,28)_	0.945	0.004
C3′	Alpha power	CONDITION	2.377_(2,28)_	0.111	0.145
(Fz reference)		TIME	2.245_(1,14)_	0.156	0.138
		CONDITION × TIME	0.554_(2,28)_	0.581	0.038
	Beta power	CONDITION	1,129_(2,28)_	0.338	0.075
		TIME	2.209_(1,14)_	0.159	0.136
		CONDITION × TIME	0.279_(2,28)_	0.758	0.020
Fz	Alpha power	CONDITION	0.628_(2,28)_	0.541	0.043
(A1 reference)		TIME	0.088_(1,14)_	0.771	0.006
		CONDITION × TIME	0.777_(1.453,20.349)_	0.435	0.053
	Beta power	CONDITION	0.523_(2,28)_	0.599	0.036
		TIME	0.627_(1,14)_	0.442	0.043
		CONDITION × TIME	0.073_(2,28)_	0.929	0.005

### Exp. 2: The Relation Between P45 Component and Alpha and Beta Power

**Figure 8:**
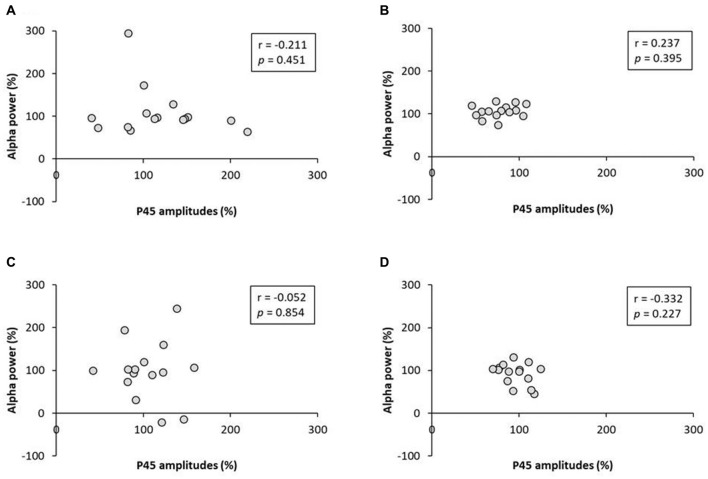
**(A)** 1.0 Hz-RPM, **(B)** 3.0 Hz-RPM, **(C)** 5.0 Hz-RPM, **(D)** control. These graphs present the relationship between the rate of change of P45 amplitudes (%) and alpha power (%) before and after RPM. No significant correlation was found between the rate of change of P45 amplitudes (%) and alpha power (%). Abbreviations: RPM, repetitive passive movement.

**Figure 9:**
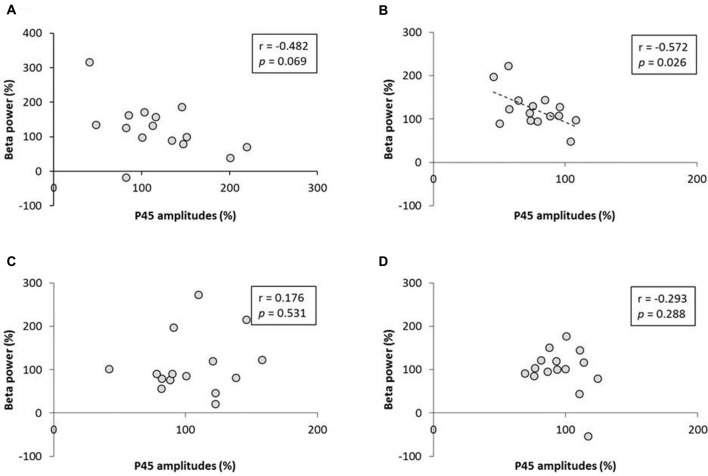
**(A)** 1.0 Hz-RPM, **(B)** 3.0 Hz-RPM, **(C)** 5.0 Hz-RPM, **(D)** control. These graphs present the relationship between the rate of change of P45 amplitudes (%) and beta power (%) before and after RPM. A negative correlation was shown only between the rate of change of P45 amplitudes (%) and beta power (%) (*r* = −0.572, *p* < 0.05) in the 3.0 Hz-RPM. Abbreviations: RPM, repetitive passive movement.

No significant correlation was found between the rate of change of P45 amplitudes and alpha power (Figure 8). A negative correlation was observed between the rate of change of P45 amplitudes and beta power in 3.0 Hz-RPM (*r* = −0.572, *p* < 0.05), but no correlation was found in 1.0 and 5.0 Hz-RPM and in the control (Figure 9).

## Discussion

This study investigated whether RPMs affect SEPs and resting EEG, including alpha and beta bands, depending on movement frequency. From our results, no significant changes were shown in N20 and P25 components before and after RPM. The P45 component decreased after the 3.0 Hz-RPM, but not after other RPMs. In addition, resting EEG, including alpha and beta frequencies, did not change before and after RPM. However, a negative correlation was found between the rate of change of P45 component and beta band for the 3.0 Hz-RPM.

In this study, we observed that the P45 component decreased after RPM. Previous studies have shown that the N20 component is produced by a tangential generator located in Brodman’s area 3b of S1 (Allison et al., 1991; Namiki et al., 1996). The P25 component has also been shown to be generated by Brodman’s areas 1 and 2 of S1, as well as area 4 of M1 (Dinner et al., 1987) or area 1 of S1 (Allison et al., 1989a). Additionally, the P45 component reflects the activity of S1 (Allison et al., 1989b, 1992; Bufalari et al., 2007). Neuroimaging studies using magnetoencephalography have reported that the source of N20m (corresponding to N20), P35m (corresponding to P25) and P60m (corresponding to P45) of somatosensory-evoked magnetic fields evoked by median nerve stimulation localize to different positions in S1 (Huttunen et al., 2006; Onishi et al., 2016). Therefore, somatosensory inputs induced by RPM may affect only specific areas in S1 because only P45 component changes have been observed.

Our study revealed that only 3.0 Hz-RPMs decreased the P45 component. Our previous study using MEPs indicated that 0.5, 1.0 and 5.0 Hz-RPMs decrease MEPs; however, no MEP changes were observed with only 3.0 Hz-RPM (Sasaki et al., 2017b). The reason for this was not clear, but it is likely that depression of the P45 component after the 3.0 Hz-RPM may disturb MEP depression. Neuroimaging studies have revealed that somatosensory input induced by passive movements without motor commands activate not only S1 but also M1 in humans (Weiller et al., 1996; Onishi et al., 2013; Piitulainen et al., 2015; Sugawara et al., 2016). In addition, somatosensory input influences M1 activity in primates via dense intracortical projections between S1 and M1 (Zarzecki et al., 1978). Additionally, TMS and EEG studies have shown that theta burst stimulation over S1 suppresses S1 excitability and facilitates M1 excitability (Jacobs et al., 2014; Tsang et al., 2014). We believe that S1 excitability depression after the 3.0 Hz-RPM through the nerve fibers from S1 to M1 acts as an enhancement of M1 excitability, and thus MEP depression was disturbed by the 3.0 Hz-RPM. In contrast, because we used four passive movement frequencies based on the findings of our previous study, we are unable to infer the results if we use other passive movement frequencies. Therefore, further study is necessary to determine if this is an accurate interpretation of these results, where the P45 component is reduced after 3.0 Hz RPM, but not after 0.5, 1.0 and 5.0 Hz-RPMs.

In Exp. 1, SEPs were recorded from the C3’ area in the Fz reference. We selected the Fz reference as this position can reduce mixing noise (Sonoo et al., 1996). However, frontal SEP components such as frontal N30 may mix for the active electrode using the Fz reference electrode (Cheron et al., 2000; Cebolla et al., 2011). Therefore, we examined whether the P45 component of SEP modulates in a non-cephalic reference in the same manner as the Fz reference. In addition, we confirmed whether the frontal N30 component of the frontal area origin was influenced by RPM intervention. Our results showed that P45 component depression was observed both with the Fz and A1 references after the 3.0 Hz-RPM. In addition, no frontal N30 changes were observed after the 3.0 Hz-RPM, and, thus, P45 component changes were caused by the C3’ area of the active electrode.

Resting EEG, including alpha and beta bands, did not change before and after RPM; however, a negative correlation was observed between the rate of change of beta power and P45 component in the 3.0 Hz-RPM. This result means that enhancement of beta power contributes to P45 component depression. Magnetoencephalography and EEG studies using time-frequency analysis have shown that alpha or beta band power is modulated by somatosensory stimulation such as peripheral nerve electrical stimulation, tactile stimulation and passive movement (Neuper and Pfurtscheller, 2001; Gaetz and Cheyne, 2006; Houdayer et al., 2006; Parkkonen et al., 2015), as well as M1 activations such as active movement and motor imagery (Cassim et al., 2000; Neuper and Pfurtscheller, 2001; Takemi et al., 2013). Additionally, sensorimotor cortex activation and deactivation are reflected in graduated changes of induced alpha and beta oscillations (Ploner et al., 2006; Hall et al., 2011; Rossiter et al., 2014), and a negative correlation has been observed between S1 excitability and the beta band (Ploner et al., 2006). Pharmacological studies have also shown that beta power increases following the administration of the GABA-A modulator diazepam (Hall et al., 2010; Premoli et al., 2017). In addition, P60m (corresponding to P45) depression appears to be caused by a GABA-A agonist (Huttunen et al., 2008). From these results, our study also showed a similar change between the beta power and P45 before and after the 3.0 Hz-RPM. Therefore, we believe that the enhancement of beta power contributes to P45 component depression after the 3.0 Hz-RPM.

Several studies have reported that transcranial alternating current stimulation, peripheral nerve electrical stimulation, and repetitive TMS affect M1 excitability in a stimulation frequency-specific manner (Fitzgerald et al., 2006; Chipchase et al., 2011; Schilberg et al., 2018); these low and high frequency stimulations modulate M1 excitability changes differently. This frequency dependent-specific manner on M1 excitability changes can be considered important to S1 excitability change using RPM, and thus S1 excitability changes induced by 3.0 Hz-RPM may depend on the RPM frequency. However, the specific S1 excitability change by 3.0 Hz-RPM cannot be entirely deduced by physiological explanations from this study. Therefore, further studies such as magnetoencephalography and multi-channel EEG are required to elucidate the effect of RPM frequency on S1 in detail.

In conclusion, we examined as to whether RPM affects SEP components and resting EEG, including alpha and beta oscillations depending on RPM frequency. Our results showed that the N20 and P25 components of SEP did not change before and after RPM, but P45 component depression was observed after 3.0 Hz-RPM but not after other RPMs. These findings suggest that somatosensory input induced by 3.0 Hz-RPM affected only the P45 component generator. There was no difference in alpha and beta bands before and after any RPM intervention. However, a negative correlation was observed between the rate of change of beta power and P45 component at 3.0 Hz-RPM, suggesting that beta power enhancement may contribute to P45 component depression. The novel findings of this study should provide new insight into the effects of RPM on the sensorimotor cortex. Additionally, these results, including our previous study (Sasaki et al., 2017b), suggest that M1 and S1 excitability for patients with motor and sensory diseases in the neurorehabilitation field may be capable of being modulated by RPM. In future, we wish to reveal the effect of neurorehabilitation by combining with motor learning task and conditioning stimulation by RPM for cortical activity.

## Author Contributions

HO and RS conceived the study, designed the experiments and wrote the manuscript. RS and ST conducted the experiments and performed statistical analysis. SM and SK performed data interpretation. KS, YI and NO helped write the manuscript. All authors read and approved the final manuscript.

## Conflict of Interest Statement

The authors declare that the research was conducted in the absence of any commercial or financial relationships that could be construed as a potential conflict of interest.
